# It's about time: the heterochronic background for the 2024 Nobel Prize in Physiology or Medicine

**DOI:** 10.1242/dmm.052187

**Published:** 2024-11-27

**Authors:** Bruce Wightman

**Affiliations:** Biology Department, Muhlenberg College, Allentown, PA 18104, USA

## Abstract

The 2024 Nobel Prize in Physiology or Medicine has been awarded to Victor Ambros and Gary Ruvkun “for the discovery of microRNA and its role in post-transcriptional gene regulation”. The award celebrates the discovery of small regulatory miRNAs and their mRNA targets, published over three decades ago. The groundwork for this discovery was laid during the early 1980s, when Ambros began studying mutations that caused heterochronic defects in the nematode *Caenorhabditis elegans* − or shifts in the temporal identities of cells. A major impetus to study the heterochronic genes of *C. elegans* was to gain mechanistic understanding of how developmental stages are specified − a fascinating question in basic and evolutionary biology. Asking fundamental biological questions with no immediate application to human health ultimately led to the discovery of a new type of RNA, which had broad implications for understanding and treating human disease.

## Teaser

**Summary:** The 2024 Nobel Prize in Physiology or Medicine has been awarded for the discovery of miRNAs; the groundwork was laid by investigating the timing of developmental events in nematodes.

The award of the Nobel Prize in Physiology or Medicine this year to Victor Ambros and Gary Ruvkun highlights a major addition to our understanding of gene regulation; the demonstration that microRNAs (miRNAs) regulate gene expression through binding to complementary sites on the mRNA of target genes (Lee et al., 1993; Wightman et al., 1993). Subsequent work demonstrated that the human genome encodes at least hundreds of miRNAs (Alles et al., 2019), that they appear to be ubiquitous among plants and animals (Pasquinelli et al., 2000; Chen and Rajewsky, 2007), and that they play important regulatory roles in various human diseases, including cancer (Trang et al., 2008; Mayr and Bartel, 2009; Ceppi and Peter, 2014). But this work didn't start out as being focused on RNA, disease or even on humans at all. The origins of miRNA discovery trace back to efforts to understand the mechanisms by which organisms coordinate temporal biological events.

When I was in my early 20s, I asked my parents for a Christmas present, Stephen J. Gould's book *Ontogeny and Phylogeny*. Gould, the great communicator of science and polymath, laid out a rich history of biological thought about the regulation of developmental stages as an important mode for evolutionary change (Gould, 1977). Although some of his claims were controversial at the time, he anticipated the field of Evolutionary Developmental Biology (EvoDevo). A key concept was heterochrony − changes in the relative timing of developmental events, particularly developmental stages. Heterochronic changes can serve as the raw material for evolutionary changes to body form and physiology. Some heterochronic changes involve later developmental events that occur at earlier stages, while others involve the extension or repetition of earlier events at later stages. For example, neoteny, first described in the 19th century, is a trait specific to axolotls, in which reproductive maturity occurs within a retained larval form. Gould recounted speculation among evolutionary biologists that heterochronic mechanisms might also explain aspects of human evolution.

In the late 1970s and early 1980s, Bob Horvitz and Martin Chalfie were leveraging the *Caenorhabditis elegans* lineage (the defined specific patterns of cell division), which Horvitz had recently described with visionary John Sulston (Sulston and Horvitz, 1977). The idea was to identify mutations in genes that perturbed these patterns of cell division in interesting ways. In 1981, they described one mutation in a gene called *lin-4* (the ‘lin’ designation identifies an effect on the cell lineage; Chalfie et al., 1981). There was nothing particularly remarkable about the appearance of the animal − it was long and flaccid, and couldn't lay eggs. But when they looked closely at cell lineage patterns, they realized that cells were actually repeating first larval stage division patterns again and again at later stages. Moreover, the adult kept molting, as if the adult skin cells believed they were still larval cells. Therefore, the functional product of the *lin-4* gene must be important for regulating the temporal identity of the cells. With *lin-4*, the first heterochronic mutation in *C. elegans* had been described. In the next few years, the Horvitz lab discovered mutations in another gene, *lin-14*, which would also prove to have heterochronic gene function (Horvitz et al., 1983).

Victor Ambros made heterochronic genes the centerpiece of his postdoctoral fellowship in the Horvitz lab (Ambros and Horvitz, 1984, 1987; Ambros, 1989). His work established a pathway of heterochronic genes, with *lin-14* as an important regulator of early larval cell identities. Mutations in *lin-14* came in two flavors: a recessive loss-of-function type that resulted in precocious larval cell division patterns and a semi-dominant gain-of-function type with the opposite phenotype (retained early division patterns at later stages), similar to *lin-4* mutants. The genetics suggested that *lin-14* was a key ‘switch’ that controlled early temporal development, with *lin-4* as an apparent negative regulator.

Gary Ruvkun joined the Horvitz lab as a postdoc a few years after Ambros, beginning the collaboration that would lead to 2024's Nobel Prize. Ruvkun and Ambros were an interesting pair: the former an outgoing, irreverent Californian, the latter a gentle, intellectual New Englander. Ruvkun set his efforts on cloning the *lin-14* gene. In the early 1980s, few genes had been positionally cloned, and the task demanded some innovation. Collaborating with early genomicists Robert Waterston (University of Washington, Seattle, WA, USA) and Alan Coulson (retired – formerly at the Sanger Centre, Hinxton, UK), they managed to identify the *lin-14* gene, demonstrating in the process that the gain-of-function mutations mapped to the 3′ end of the gene−the first hint that there might be something interesting about *lin-14* regulation (Ruvkun et al., 1989). There was just one problem − the sequence of the encoded LIN-14 protein bore no resemblance to any known protein (Wightman et al., 1991). Ruvkun once gave a talk in which he showed a slide with a single empty box labeled “region of the *lin-14*-encoded protein with no similarity to any known protein.” It seemed that the study of heterochronic genes might be relegated to the backwaters of nematode esoterica.

In 1985, Ambros and Ruvkun took faculty positions across Boston's Charles River from each other (Harvard and Massachusetts General Hospital, respectively) and continued to collaborate on the heterochronic pathway. When I landed in the Ruvkun laboratory as a Ph.D. candidate in 1987, I entered a world of vibrant, youthful intelligence, a tennis ball-chewing dog and of free espresso. It was a stimulating environment, to say the least. Like many graduate students, I spent a couple of years flailing around on a project that didn't go much of anywhere. Finally, I got my chance to work on heterochronic genes. By then, Ruvkun had shown that the LIN-14 protein is expressed in a temporal gradient*,* exactly as you might expect from Ambros's genetic analysis (Ruvkun and Giusto, 1989). Postdocs Thomas Bürglin and Prema Arasu, technician Joe Gatto, and I showed that *lin-14* was regulated post-transcriptionally and depended on *lin-4* (Wightman et al., 1991; Arasu et al., 1991). In collaboration with postdoc Ilho Ha, I set my attention on the *lin-14* 3′-untranslated region, identifying evolutionarily-conserved blocks that were required for the temporal regulation of protein expression (Wightman et al., 1993).

**Figure:**
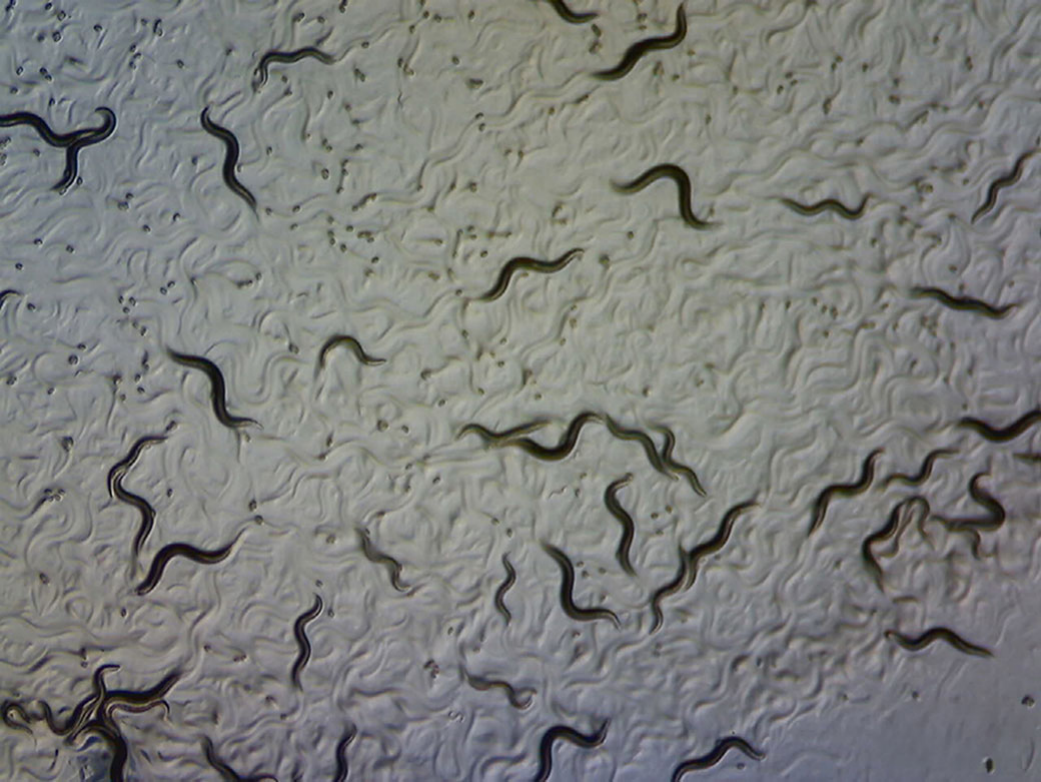
***Caenorhabditis elegans* eggs, larvae and adults.** The image is licenced under a Creative Commons Attribution 4.0 International license.

Meanwhile, over at the Cambridge campus of Harvard University, Rosalind Lee (University of Massachusetts Chan Medical School, Worcester, MA, USA) and Rhonda Feinbaum (Massachusetts General Hospital, Boston, MA, USA) in the Ambros lab were doing the arduous work of identifying a new *lin-4* mutation and cloning the gene. It proved to be the ‘incredibly-shrinking gene’, as they found smaller and smaller fragments of DNA that contained *lin-4* functional activity (Lee et al., 1993). Through careful analysis, Lee and Feinbaum ruled out the possibility this being a small peptide, and defined what would come to be called an miRNA (Lagos-Quintana et al., 2001). In a cinematic scene, Ambros and Ruvkun read the sequence of the *lin-4* RNA and my *lin-14* 3′UTR blocks to each other over the phone, leading to the eureka moment when realizing that *lin-4* RNA regulates *lin-14* temporal expression by RNA-pairing (Ruvkun et al., 2004; Lee et al., 2004).

When we published the work in back-to-back *Cell* papers in 1993 (Wightman et al., 1993; Lee et al., 1993), not everyone was as excited as we were. Vertebrate researchers with an interest in RNA and gene regulation, such as Marv Wickens (retired – formerly University of Wisconsin, Madison, WI, USA) and Kathy Takayama (Brown University, Providence, RI, USA), thought this pointed to something much larger (Wickens and Takayama, 1994); but the problem remained that both *lin-4* and *lin-14* seemed to be peculiar to worms. The persuasive studies would come later, as members of the Ruvkun and Ambros labs, as well as the lab of David Bartel (Massachusetts Institute of Technology, Cambridge, MA, USA), demonstrated that there were many miRNAs, and that they were also found in plants and vertebrate animals (Pasquinelli, et al., 2000; Lee and Ambros, 2001; Lim et al., 2003). Among these was *let-7*, now implicated in a range of important regulatory functions related to human disease (Reinhart et al., 2000; Pasquinelli et al., 2000; Ambros, 2004; Esquela-Kerscher and Slack, 2006).

Throughout the 1990s and to this day, work on the heterochronic pathway of *C. elegans* has continued, with many genes now identified, and their complex regulatory interactions and functions described (Ambros and Moss, 1994; Slack and Ruvkun, 1997). Among them is *lin-28*, another target of *lin-4* miRNA, which encodes an RNA-binding protein. *lin-28* is evolutionarily conserved and seems to play a role in regulating the onset of mammalian puberty (Cao et al., 2020). Therefore, at least one *C. elegans* heterochronic gene appears to be predictive for understanding mammalian development − and, potentially, human disease − by more conventional orthology and functional conservation. Recent work has also shown that *lin-14* has a structural relationship to a family of conserved transcription factors (Greene et al., 2023), so perhaps it, too, isn't quite the orphan we thought.

miRNA genes have been there for hundreds of millions of years, awaiting discovery. They would, eventually, have been found one way or another, but the history of this discovery started with asking mechanistic questions about animal development in a microscopic nematode. In contrast to the textbook view of science, there was no specific hypothesis being tested. We can't propose a function for miRNAs until we know they are there. This history is more about leveraging the power of new technologies on questions of biological significance – it's discovery-based science. There is a lesson here in how we prioritize and fund science. Francisco Mojica's report of repeated sequences in bacteria in 1993 didn't get a lot of attention (Mojica et al., 1993), yet was the first step in a research program that, eventually, led us to CRISPR gene editing. Of course, not every odd little corner of biology will lead to something broadly significant. But new discoveries often emerge from unexpected places. When my students, who are headed to graduate school, ask me about a thesis strategy, I tell them: first and foremost, identify a great mentor. Second, find an interesting problem that is technically tractable, no matter how strange it might seem, and run with it. After a few decades, who knows?
